# Integrated near-infrared spectral sensing

**DOI:** 10.1038/s41467-021-27662-1

**Published:** 2022-01-10

**Authors:** Kaylee D. Hakkel, Maurangelo Petruzzella, Fang Ou, Anne van Klinken, Francesco Pagliano, Tianran Liu, Rene P. J. van Veldhoven, Andrea Fiore

**Affiliations:** Department of Applied Physics and Eindhoven Hendrik Casimir Institute, Eindhoven University of Technology, PO Box 513, NL 5600 MB Eindhoven, The Netherlands

**Keywords:** Optical sensors, Sensors

## Abstract

Spectral sensing is increasingly used in applications ranging from industrial process monitoring to agriculture. Sensing is usually performed by measuring reflected or transmitted light with a spectrometer and processing the resulting spectra. However, realizing compact and mass-manufacturable spectrometers is a major challenge, particularly in the infrared spectral region where chemical information is most prominent. Here we propose a different approach to spectral sensing which dramatically simplifies the requirements on the hardware and allows the monolithic integration of the sensors. We use an array of resonant-cavity-enhanced photodetectors, each featuring a distinct spectral response in the 850-1700 nm wavelength range. We show that prediction models can be built directly using the responses of the photodetectors, despite the presence of multiple broad peaks, releasing the need for spectral reconstruction. The large etendue and responsivity allow us to demonstrate the application of an integrated near-infrared spectral sensor in relevant problems, namely milk and plastic sensing. Our results open the way to spectral sensors with minimal size, cost and complexity for industrial and consumer applications.

## Abstract (web summary)

What are the minimal hardware requirements for a given class of sensing problems? Here, authors investigate this while proposing a miniaturized near-infrared spectral sensor, based on an array of resonant-cavity enhanced photodetectors, and capable of operating without the need for spectral reconstruction.

## Introduction

Optical spectroscopy has been used for decades for quantifying the chemical composition of objects with dimensions ranging from a few nanometers to millions of kilometers. Recently, the need for in-situ and on-the-field sensing has been driving the quest for spectral sensors with reduced footprint, increased portability and lower cost, suitable for consumer applications and embedding into smartphones^1–4^, and several portable spectroscopy products have reached the market (see Ref. ^4^ for an overview). Chip-level integration and mass manufacturing using semiconductor processing methods are required for this purpose. While substantial progress has been made in the visible spectral region and up to 1100 nm, using the high maturity of silicon processing^2,3^, progress in the integration of near-infrared (NIR) spectrometers has been limited. The near-infrared part of the electromagnetic spectrum, and in particular the region 1000–1700 nm, is especially interesting for spectroscopy of organic materials, due to the presence of absorption bands arising from overtones and combinations of vibrational modes of O-H, C-H, and N-H bonds. NIR spectrometry provides higher sensitivity than visible solutions for important application cases in agri-food and health care^5,6^. Integration of NIR spectrometers has been mainly pursued using dispersive waveguide geometries^7–11^ and the combination of micro-electromechanical (MEMS) filter structures co-packaged^12–14^ or integrated with detectors^15^. However, nearly all applications use diffuse transmittance or reflectance, involving spatially-incoherent light, which prevents the use of single-mode waveguide structures. On the other hand, MEMS approaches are affected by sensitivity to mechanical vibrations and make monolithic integration with NIR detectors challenging.

**Fig. 1:**
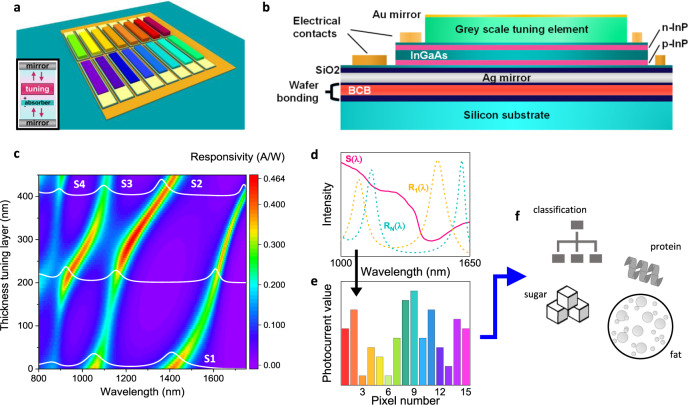
Spectral sensing mechanism of a resonant-cavity-enhanced (RCE) multi-pixel array. **a** Top view sketch of a multi-pixel array where each pixel (indicated by the different colors) has a different wavelength response. Inset: Sketch of an RCE detector, where both the absorber and the tuning element are positioned within the vertical-cavity structure. **b** Cross section of a single RCE detector (not to scale). **c** Color plot of the simulated responsivity for different thicknesses of the tuning layer. Spectra for 0, 200, 400 nm thickness are shown in white. S1 to S4 indicate the different resonances. **d** Sketch showing the filter responses ***R***_***i***_(***λ***) (as calculated for two different tuning layer thicknesses) together with the incident light spectrum ***S***(***λ***) (smoothened experimental transmission spectrum of raw milk). **e** Schematic illustration of the generated photocurrent values for 15 pixels following Eq. 1, which can be used **f**, directly for spectral sensing, e.g., to classify or quantify properties of the measured sample.

In general, integrating a spectrometer on a chip is exceedingly difficult. However, the goal of spectral sensing is not the measurement of a spectrum, but rather the characterization of the material which has interacted with light. Human eyes are excellent spectral sensors, providing essential information on food, health condition, and material properties, based on the signals from only three types of cone cells, which have broad and overlapping spectral responses. Other animals have developed more advanced multispectral viewing systems, involving up to 16 different filters^16^. This motivates us to ask the question: What are the minimal hardware requirements for a given class of sensing problems? In general, spectral sensing is based on the measurement of the light emitted, transmitted, or reflected by an unknown material employing a sequence of filters. This can be done wavelength-by-wavelength (grating spectrometers), in the Fourier domain (Fourier-transform spectrometers), or in any other basis (e.g., computational spectrometers based on chaotic filters^7,11^). The use of generalized filters can in fact greatly simplify their on-chip integration and has been investigated^17–23^. Given the responses of the filters and the expected spectral characteristics of the sample, the input spectrum can be reconstructed and chemometric approaches are then applied for the intended classification or quantification goal (Fig. 1d–f).

Here we report a NIR spectral sensor, which is suitable for diffuse transmittance/reflectance measurements, is fully integrated, and has no moving parts. It is based on a small array of detectors with distinct spectral responses. We then build a predictive model directly based on the photocurrent data. Our approach is motivated by the observation that the intermediate step of spectral reconstruction is not needed—the signals from the detectors can be directly used to train a model for the sensing problem at hand, in the same way as the signals from cone cells are used to train human perceptive abilities. This observation greatly simplifies the requirements on the hardware, leading to a simple and compact sensor design.

## Results

### Design of a NIR multi-pixel array for spectral sensing

In pursuit of a simple integrated filter-detector design, we investigate the use of a simple set of resonant-cavity detectors as a suitable platform for spectral sensing. We developed a fully-integrated NIR sensor based on an array of resonant-cavity-enhanced (RCE) photodetectors operating in the 850–1700 nm wavelength range (see Fig. 1a).

Each pixel of the array (Fig. 1b) contains a thin absorbing layer positioned inside a Fabry–Perot (FP) cavity, resulting in a strong spectral dependence of the quantum efficiency *η*_*L,R*_(*λ*)^24^. This spectral response is controlled for the pixels individually by changing the length of each FP cavity via a tuning element inside the cavity (see inset Fig. 1a). This provides direct integration of the filters and the detectors in a single robust device, eliminating alignment errors and the need for micromechanical tuning. Besides, the RCE structure allows reaching high efficiencies with a thin active region and low dark current^25^. The active layers consist of a p-i-n InP photodiode with a 200 nm InGaAs absorber layer. A SiO_2_ spacer layer is positioned in between the bottom Ag mirror and the active layers in order to improve the absorption in the InGaAs layer. The tuning layer is made of a dielectric layer with a 10-nm thick semi-transparent Au mirror on top. Additional metal layers employed for the n- and p- contact of the photodiode are positioned outside the cavity region on top of two corresponding InGaAs contact layers. Two 100-nm thick InP barriers separate these contact layers from the InGaAs absorber layer.

The optical response is evaluated using finite difference time domain (FDTD) simulations (see Methods). Figure 1c shows the calculated responsivity when the tuning layer is varied from 0 to 450 nm. The response curves of the detectors show four distinct broad peaks, attributed to the different FP modes (S1, S2, S3, and S4). When increasing the thickness of the tuning layer, all modes shift to higher wavelengths, but not with the same rate, due to their different spatial field distributions (see Methods and Supplementary Fig. 1). Mode S2 shows the largest tuning range of 370 nm when the tuning layer thickness is increased from 0 to 450 nm. Full coverage of the NIR range (850–1680 nm) is obtained using multiple modes. The different modes are characterized by a full width at half maximum (FWHM) of ~50–100 nm with corresponding Q-factors λ/Δλ between 10 and 30 and a maximum responsivity of ~0.13–0.46 A/W. The interaction between the different resonant modes causes the observed avoided crossings and the variation in the FWHM. Additional simulations show that increasing the reflectivity of the top mirror suppresses the background absorption and results in sharper peaks, at the expense of lower responsivity^24^ (see Supplementary Fig. 2a). As expected for a planar cavity, the angular dependence of the resonant wavelength is low (max ~20 nm for 20°, see Supplementary Fig. 2c), as compared to plasmonic filters^26^ or photonic crystal slabs^21^, which is a major advantage for efficient light coupling.

### Realization of the multi-pixel array

**Fig. 2:**
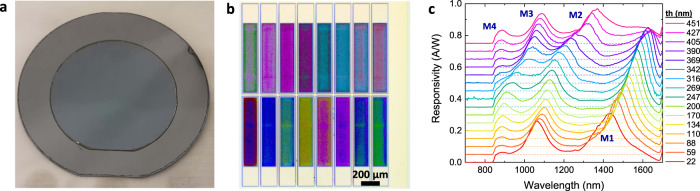
Realization of a multi-pixel array. **a** 2″ InP membrane integrated on a Si wafer via adhesive wafer bonding. **b** Optical microscope image of a detector array (magnification 5x). **c** Measured responsivity for 16 pixels of the same array with measured Ma-N thickness increasing from 22 to 451 nm. Resonant modes are indicated by M1 to M4. An offset, with increasing steps of 0.05 A/W, is added for clarity. The zero-responsivity axis for each curve is indicated by the dashed line in the corresponding color.

We realized the proposed sensors, where an array contains 16 pixels, each with 150 μm × 750 μm active area and varying height. During the fabrication process (see Methods and Supplementary Fig. 3) the epitaxially grown InP wafer is bonded on a silicon wafer via adhesive wafer bonding (see Fig. 2a). This hybrid integration approach, known as InP-membrane-on-silicon (IMOS)^27^, allows for double-side processing of the active layers, key for the fabrication of the bottom mirror. Importantly, to introduce the wavelength tuning of the photodiodes, the three-dimensional height of the dielectric tuning layer (ma-N resist) is simultaneously varied for all pixels in a single step using grayscale lithography^28^. Figure 2b shows an optical microscope image of a fabricated detector array, displaying different colors arising from spectral resonances in the visible region related to the varying pixel height. The non-uniformity observed within the pixel is a result of a non-optimized proximity effect correction employed in the grayscale electron beam lithography (see Methods).

Figure 2c shows the measured responsivity of 16 pixels with tuning layer thickness varying from ~20 to ~450 nm, as measured with a focused illumination spot and a two-finger probe (see Methods). Several peaks can be identified (M1, M2, M3, and M4) and associated with the modes (S1 to S4 in Fig. 1c) expected from simulation. Mode M2 shows a redshift of 310 nm in the measured tuning range for a ma-N tuning layer thickness increase from 22 to 451 nm, which is in the same order as the 350 nm shift expected from simulations (see Methods and Supplementary Fig. 4a). The maximum measured responsivity is ~0.36 A/W for mode M1 and 0.26 A/W for mode M2. The FWHM of M2 varies between ~100 and 210 nm, with corresponding Q-factors between 5 and 15, as determined via multiple Lorentzian peak fitting. This higher FWHM and deviations in the responsivity as compared to simulations are attributed to the surface roughness of the tuning layers and can be improved by further optimizing the fabrication process. The decreased photoresponse below ~950 nm is caused by the increased absorption in the InP layers below this wavelength. The sharp responsivity cut-off below 840 nm is due to the long-pass filter used in the characterization setup and the small ripples present on top of the spectra are attributed to the reflections in the optical measurement system.

### Application in spectral sensing

The mechanism of spectral sensing using multiple pixels is illustrated in Fig. 1d–f. During sensing operation, the detector array is illuminated with an unknown spectrum *S*(*λ*), resulting in a generated photocurrent *I*_*i*_ for each pixel *i*, depending on its responsivity *R*_*i*_(*λ*),

1 $${{{{{{\boldsymbol{I}}}}}}}_{{{{{{\boldsymbol{i}}}}}}}={\int }_{{\kern-0.4pc{{{{{\boldsymbol{\lambda }}}}}}}_{1}}^{{{{{{{\boldsymbol{\lambda }}}}}}}_{2}}{{{{{\boldsymbol{S}}}}}}({{{{{\boldsymbol{\lambda }}}}}}){{{{{{\boldsymbol{R}}}}}}}_{{{{{{\boldsymbol{i}}}}}}}({{{{{\boldsymbol{\lambda }}}}}}){{{{{\bf{d}}}}}}{{{{{\boldsymbol{\lambda }}}}}},({{{{{\boldsymbol{i}}}}}}=1,2,\ldots ,{{{{{\boldsymbol{N}}}}}})$$

where *λ*_*1*_ and *λ*_*2*_ define the operating wavelength range of the sensor and *N* is the number of detector pixels. The incident light spectrum can be retrieved by inverting this set of equations^20,23,29^, using the knowledge of *R*_*i*_(*λ*), and then used to determine the target material property (e.g., fat concentration) using standard chemometric methods. However, the step of spectral reconstruction requires making assumptions on the spectrum to be measured, is prone to numerical errors, and artificially expands the set of spectral data, which is then compressed again by the chemometric analysis into a limited number (5–10) of spectral components. Here we instead show that photocurrent data from the detector array can directly be used as input to train a quantification or classification model (blue arrow in Fig. 1). This requires no assumptions on the spectra and uses the available spectral information optimally.

**Fig. 3:**
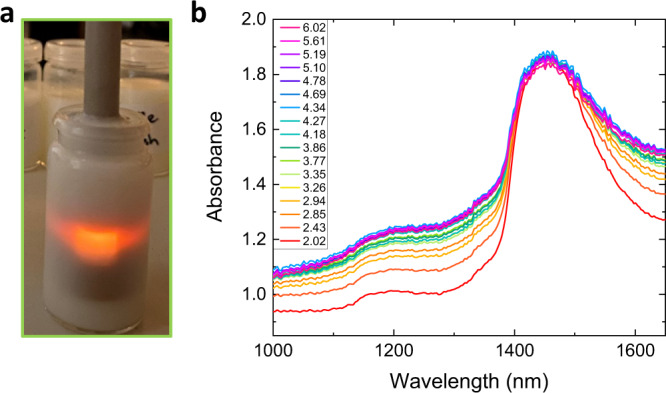
Spectral sensing of milk samples. **a** Transflection probe inserted in a milk sample. **b** Absorbance of 17 milk samples with different fat wt%, measured using a commercial spectrometer.

As a proof of principle, we present here the use of the multi-pixel array for the measurement of the nutritional properties of milk—a sensing problem of practical relevance as it impacts the economic value of milk and it helps monitor the cow’s health^30^. In this experiment, commercially available raw milk standards were used, containing calibrated fat concentrations varying between 2.02 and 6.02 g/100 g. The samples were probed using a transflection probe (see Fig. 3a). We note that the multimode nature of the transflection probe (300 μm core diameter) would prevent the use of integrated spectrometers based on waveguides^31^, chaotic structures^7^, or nanophotonic cavities^15^, for this and any other practical diffuse transmittance application. Figure 3b shows the absorbance spectra, measured with a commercially available mini spectrometer (see Methods), with a strong water absorption peak around 1450 nm. The variation with fat percentage is non-monotonic, as is clearly visible around the absorption peak where the maximum absorbance is obtained for a fat percentage around 4.5%, highlighting the need for a spectral sensor. As commonly observed in NIR sensing problems, the spectral features are broad, indicating that high spectral resolution is not required.

**Fig. 4:**
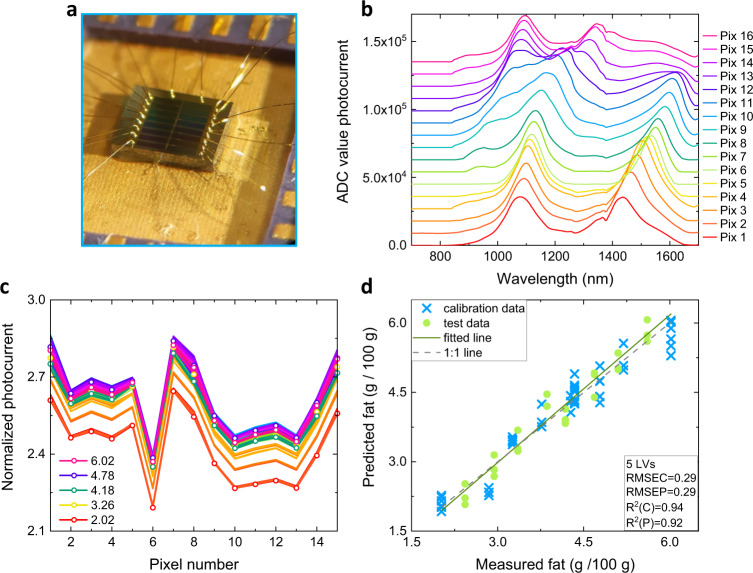
Spectral sensing of milk samples. **a** Wire-bonded array for single-shot readout. **b** Response curves of wire-bonded pixel array for sensing. ADC photocurrent values were measured with the developed amplifier board as a function of wavelength of illumination (linewidth ~5.5 nm, power ~1.95 μW). An offset with increasing steps of 9000 ADC between the response curves of different pixels is added for clarity. **c** Scaled absorbance values of selected raw milk samples measured for the 15 RCE pixels. **d** The fat content predicted via PLS regression using normalized photocurrent values from the RCE detector compared to the expected fat content. The number of latent variables (LVs), the root mean square error of calibration (RMSEC) and prediction (RMSEP), and the coefficient of determination for both the calibration R^2^(C) and prediction R^2^(P) are indicated.

We developed a custom amplifier board containing sixteen trans-impedance amplifiers, an ADC multiplexer and a microcontroller to read out the photocurrent array in a single-shot measurement (see Fig. 4a and Methods). We characterized the dynamic range of the sensor over five orders of magnitude using the electronic board for readout (see Supplementary Fig. 5). Input powers down to the pW level can be measured and the minimum detectable power is limited by the electronics of the readout board. One pixel was used to correct for the drift introduced by the used amplifier board and the remaining 15 pixels for the transflection measurements (see Methods). Figure 4b shows the response curves in ADC units, as measured with the setup for simultaneous readout. Note that for these sensing experiments we used a multi-pixel array different from the one characterized in Fig. 2c, as contacts pads are damaged by the probes used for the experiment in Fig. 2c. Figure 4c shows the scaled absorbance for the different milk samples for each pixel, where each pixel has a broadband optical response. The 15 scaled absorbance values from each measurement of the calibration sample set were mean-centered and, after outlier elimination, directly used in the prediction model (see Methods). Partial least squares (PLS) analysis was applied to reduce the multidimensional pixel data to latent variables (LVs), from which a regression model was built for the prediction of fat content. The prediction performances were evaluated using the coefficient of determination (*R*^2^) and the Ratio of Performance to Deviation (RPD) (see Supplementary Table 1). An RPD value above three is generally accepted as a properly working prediction model^32^. Interestingly, only five LVs are required to give *R*^2^ = 0.92 and an RPD value of 3.57 (see Fig. 4d), indicating an accurate prediction model.

The prediction accuracy can be compared to the analysis performed on the full spectra measured by a commercial mini spectrometer (Fig. 3b). PLS was also applied to analyse the multidimensional spectral data (see Methods). Using all 696 wavelength points obtained together with three LVs gives an *R*^2^ = 0.94 and an RPD value of 4.05. Here no sophisticated pre-processing was performed on the spectra (e.g., smoothing), so this prediction performance should only be seen as an indication. While higher prediction performance has been reported for fat in milk using bulky spectrometers^30^, the prediction accuracy of the fat content obtained from our sensor measurements is close to values reported with commercially available NIR range centimeter-scaled systems^33^.

## Discussion

Our array allows accurate predictions of the fat content in milk, despite providing a much lower number of measured variables compared to conventional spectrometers. This is related to the absence of sharp spectral features in the measured spectra, which is also confirmed by the spectrally broad LVs which result from the PLS analysis on the measured full spectra (see Supplementary Fig. 8). We expect that similar sensing performance can be obtained for a wide range of organic and inorganic materials. We indeed show in Supplementary Discussion 1 that the same approach can successfully be applied to a different case, namely the classification of different types of plastic. 48 samples were evaluated, belonging to four plastic types: 1 (PET), 4 (LDPE), 5 (PP), and 6 (PS). Using an optimized PLS-LDA (linear discriminant analysis) model, we obtained 89.6% accuracy in cross-validation of the calibration dataset and 94.9% in prediction of the test dataset. Moreover, optimization of the technology and of the pixel design can further improve the prediction results. In order to confirm this, we performed a series of simulations utilizing a more comprehensive dataset of milk transmittance spectra from ref. ^30^. The expected photocurrents for a given sensor design were calculated from Eq. (1) using the simulated responsivity curves and used to train a prediction algorithm (see Supplementary Discussion 2). A particle swarm optimization applied to the tuning layer thicknesses leads to an RPD of 7.63 and *R*^2^ = 0.98 using 15 pixels with the same geometry and layer stack of Fig. 1b. The strong improvement is attributed to the reduction of the resonant line widths in combination with an optimized set of tuning layers for the specific sensing problem. To further prove the generality of the approach, a similar analysis is performed for a different sensing problem, the measurement of sugar content in tomatoes (see Supplementary Discussion 3), showing the ability of the detector array to perform accurate sensing predictions based on more complex spectral signatures. Calculating the expected photocurrents using the measured filter responses of Fig. 4b results in a prediction model with an *R*^2^ of 0.92 and RPD value of 3.83 while optimizing the tuning layer thicknesses for this specific application further improves the prediction accuracy with an *R*^2^ of 0.95 and RPD value of 5.09. While the optimum sensing performance is in principle obtained by matching the spectral filters to the LVs of the spectra, an approach known as multivariate optical computing^34^, our optimization method allows keeping the exact same cavity structure and avoids complex filter structures. In addition, a reduction of the linewidth will decrease the spectral overlap between adjacent pixels and thereby the correlation between the generated photocurrent values, which will enhance the prediction performance at a comparable signal to noise levels. Finally, while the presented detector array is designed to operate in the NIR range up to 1.7 µm, this range can be extended into the mid-infrared (up to 2.5 µm) by changing the absorber material, e.g., using extended InGaAs^35^, InGaAsSb^36^ alloys or to even higher wavelengths (up to 10 μm) when using type-II superlattices based on InAs/GaSb or InAs/InAsSb^37^ superlattices. When extending to the mid-infrared range a different tuning layer should be used which is suitable for greyscale lithography and transparent in the mid-infrared e.g. hydrogen silsesquioxane (HSQ)^28,38–40^.

In conclusion, RCE multi-pixel arrays represent a major step in the miniaturization of spectral sensors. Their size, comparable to a smartphone camera image sensor, together with the ease of fabrication and robust structure enables its use in portable sensing applications. Most components that can be detected by commercially available spectrometers such as sugar, starch, fat, protein, etc., present relatively broad spectral signatures in the NIR and might also be detected using an array-based spectral sensor optimized and tailored to the application. Optimization of the linewidth and reduction of the active pixel area would allow further increasing the resolving power for more demanding sensing applications. The sensors may be applied in many areas where NIR spectroscopy has demonstrated its potential, such as precision agriculture, clinical settings, personalized health care, lab-on-chip diagnostics and can ultimately be integrated into smartphones for consumer applications.

## Methods

### FDTD simulations of the resonant-cavity enhanced photodetectors

The optical responses of the resonant-cavity enhanced (RCE) devices were evaluated using finite difference time domain (FDTD) simulations. A two-dimensional representation of the structure was used in combination with periodic boundary conditions in the in-plane direction to reduce the simulation space. The refractive index of the photoresist layer (ma-N, Micro Resist Technology) is equal to *n* ≈ 1.61 in the wavelength range of interest. The imaginary part of the refractive index has been obtained via FTIR measurements giving *k* ≈ 0.0006 over the full range. The quantum efficiency (*η*), assumed equal to the calculated absorptance, is used to determine the responsivity using

2 $$R=\eta \frac{q}{h\nu }$$

where *q* is the electron charge, *ν* the optical frequency, and *h* Planck’s constant. In this calculation, the assumption is made that every photon is converted into an electron-hole pair.

Increasing the thickness of the tuning layer introduces a redshift of the resonant modes. The different modes indicated in Fig. 1c, are characterized by a full width at half maximum (FWHM) of ~50–100 nm and a maximum responsivity of ~0.13–0.46 A/W. For a 200 nm InGaAs layer on an InP substrate in the air, the responsivity is below 0.18 A/W everywhere in the 850 to 1600 nm range. Forming an RCE structure instead introduces a wavelength selectivity of the optical responses with peak absorptance that can exceed the value corresponding to a single InGaAs layer with the same thickness, while suppressing the background response. The observed responsivity values of our spectral sensor do not equal the highest responsivities reported for RCE detectors^41^, however, the chosen approach allows for tuning across the full NIR range. It should also be noted that the optical responses are spectrally broad and therefore have an overlapping response curve, resulting in correlated photocurrent values for adjacent pixels. The observed variations in the FWHM Δλ and corresponding Q factor are explained by varying spatial distribution of the modes. When the overlap between the E-field distribution of the resonant mode and the InGaAs layers (including the contact layers) is the highest, the largest FWHM is obtained. The electric field (E-field) distribution across the structure determines the amount of tuning. Supplementary Fig. 1 shows the total electric field distribution of modes S2 and S3 for three different thicknesses of the tuning layer and a 10 nm Au top mirror. The calculations show that the largest wavelength tuning of the resonant modes as a function of tuning layer thickness is observed when most of the E-field is located in the ma-N layer (see Supplementary Fig. 1b, e), whereas the observed tuning is minimized for E-field being equally distributed between the ma-N and the active layers (see Supplementary Fig. 1a, f). Besides, the number of lobes within the active layers increases for higher mode orders.

The spectral width and the peak responsivity of the optical modes are influenced by the reflectance of the semi-transparent top mirror. Supplementary Fig. 2a shows the calculated responsivity curves for the thickness of the Au top mirror varying between 5 and 40 nm for a structure with a 300 nm tuning layer. While increasing the thickness of the top mirror, and thereby its reflectance, the resonances become sharper and the background is more suppressed. However, this goes together with a decrease in the peak responsivity, since less light is coupled to the resonance when the cavity is under coupled. The reflectance of the Au mirror for a wave incident from the ma-N layer with varying thickness of the Au layer is shown in Supplementary Fig. 2b. For a 10 nm-thick mirror the maximum reflectance of the mirror is close to 74% for the highest wavelength around 1700 nm, while its value decreases to just 26% for a wavelength of 700 nm. In this work, we have chosen a 10 nm-thick Au mirror, to ensure a high responsivity and thereby an adequate signal-to-noise ratio (SNR) for the sensing experiments.

Use in a real sensing application requires a large tolerance of the RCE detector arrays in terms of angular dependence. Indeed, since the collection area on the measured sample is in general larger than the area of the used pixel array, a lens will be used to focus the light on the detectors. Supplementary Fig. 2c shows the spectral responses of a device with a 450 nm tuning layer for different angles of the incoming light with respect to normal incidence. The maximum angular dependence is observed for mode S2, showing a ~20 nm blue shift for a 20° change in the angle (a typical angular aperture in a compact sensing system), which is much less than the FWHM (~70 nm) of this mode. For an incoming angle of 40°, the wavelength shift (65 nm) becomes comparable to the FHWM of mode S2.

### Device fabrication

Fabrication of the device starts with the epitaxial growth of the active layers on an undoped InP wafer by metalorganic vapor-phase epitaxy (MOVPE). Zn doping is used for the p-layers (1.6 ∙ 10^19^ cm^−3^ for InGaAs and 10^18^ cm^−3^ for InP) and Si doping for the n-layers (10^19^ cm^−3^ for InGaAs and 5 ∙ 10^18^ cm^−3^ for InP). After the removal of an InP capping layer, the SiO_2_ spacer layer is deposited on top of the InGaAs contact layer using plasma-enhanced chemical vapor deposition. Evaporation is used to fabricate the bottom Ag mirror on top of the active layers (see Supplementary Fig. 3a). The Ag is covered on both sides with 2 nm evaporated Ge promoting the adhesion to the SiO_2_ layers. After the metal evaporation, the sample is annealed at 350 °C for 30 s to minimize defects in the mirror. To promote the adhesion to the adhesive polymer during wafer bonding, SiO_2_ layers are deposited on both the InP and the Si wafer. The adhesive polymer benzocyclobutene (BCB) is spin-coated on the Si wafer and used to glue the two parts together. The two wafers are brought in contact (see Supplementary Fig. 3b) and then bonded under vacuum at high temperature (280 °C) using a high force (340 N). After the bonding process, the substrate is removed via wet-etching leaving the n-doped InGaAs layer exposed on the top.

After this a mesa (180 μm × 980 μm) is etched down to the bottom p-doped InGaAs layer, using a SiN hard mask, patterned using electron beam lithography (EBL), in combination with several wet-etching steps. The p-doped InGaAs layer is not etched, in order to leave a common p-contact layer for all pixels (see Supplementary Fig. 3c). To electrically access the array, the p- and n- contacts are evaporated on the doped InGaAs layers and combined with a lift-off process using the pattern defined by EBL (see Supplementary Fig. 3d). To obtain ohmic contacts, a Ti/Pt/Au (25/75/200 nm) metal stack is used for the p-layer and a Ni/Ge/Au (30/50/250 nm) stack for the n-layer. Both contacts are positioned outside the optically sensitive region (150 μm × 750 μm) to separate the electrical and optical properties. At this point of the process, the optical responses of all pixels of a single array are identical. However, there is already a wavelength-dependent response of the detectors since a weak cavity is formed between the bottom Ag mirror and the top n-InGaAs/air interface.

The response is tuned by changing the cavity length of each pixel independently. A negative resist layer (ma-N 2410) is used as a tuning layer since it can be directly used for grayscale lithography. During the EBL exposure, the dose is increased stepwise for the different pixels, resulting in thicker layers for higher doses after the development of the resist. During the exposure, a three-dimensional proximity effect correction (PEC) is used to correct for the observed bowing of the pixel surface when no PEC is applied. However, the used PEC also causes roughness of the ma-N layer with height variations of ±15 nm. Further optimization of the correction algorithm is needed to solve this roughness issue. Besides, optical lithography can be used instead, resulting in more uniform pixels and thereby improved optical characteristics. A final EBL step is used to define the top Au mirror (see Supplementary Fig. 3e), using metal evaporation in combination with a lift-off process.

The overall size of the sensor is currently limited by the size of the contact pads for electrical readout. We are making use of wire-bonding of the electrical contacts to read out all pixels simultaneously, for which a minimum contact pad size is required (~100 μm width for our current equipment). Besides, in the current design, the pixel size is optimized in such a way that the pixels form a square array that has a high overlap with a circular illumination spot. Using 16 pixels provides good coverage of the NIR spectral region of interest with filter line widths in the 50–100 nm range. The pixel area can be easily reduced to a few tens of μm, which, together with narrow linewidth and flip-chip bonding, would allow a higher spectral resolution to be achieved in a comparable chip area. We speculate that the optimal trade-off between spectral resolution and signal-to-noise ratio will depend on the sensing problem.

### Single-pixel response function characterization

In the initial characterization measurements, the spectral responsivities *R*_*i*_(*λ*) of each pixel were individually measured. A fiber-coupled 20 W tungsten light source (average output power of 8.8 mW) was used for illumination. The light was filtered by a monochromator resulting in output spectra with the wavelength tuned from 800 to 1700 nm and a full width at half maximum (FWHM) of ~5.5 nm and ~2.5 µW output power. The filtered light was focused (~80 µm spot) on the pixel using a 50x objective (0.45 NA) with a ~4% coupling efficiency. An electrical two-finger probe was used for the electrical readout of the photocurrent while the wavelength of the incoming light was varied. The photocurrent values were converted to responsivity using the power of the illumination spot as measured using an external power meter.

### Comparison measured response curves with simulation

For the comparison of the experimental responsivities to the simulated values, the heights of the fabricated tuning layers were measured using a profilometer and used as input in the simulation. This thickness measurement was performed on an array, which had the same dose factors as the ones of the device used in the optical characterization while being located on a different part of the sample. Supplementary Fig. 4a shows the responsivity for the pixel with tuning layer thicknesses increasing from 22 to 451 nm. Peaks T1, T2, T3, and T4 can be directly related to the modes M1 to M4 in the measured spectra. Mode T2 shows a redshift of 350 nm for an increase from 22 to 451 nm of the tuning layer. The FHWM of the peaks varies between 50 and 100 nm, which is lower than the value observed in the measured spectra. The maximum responsivity is ~0.36 A/W for mode T1 and 0.45 A/W for mode T2. In the measured response curves, the highest responsivity of mode M2 is observed for the thinnest tuning layer, indicating that the optical performance is the least impaired for the thinnest ma-N layer. For thicker ma-N layers, the difference in the simulated and measured resonant wavelengths also increases, indicating a deviation in the dispersion of the ma-N layer used in the simulations. It should be noted that the ma-N material properties also depend on the dose used in the grayscale lithography processes. Supplementary Fig. 4b–d show that indeed the comparison between simulation and experimental response curves is the most accurate for the thinnest ma-N layer. The discrepancies between simulation and experimental results in terms of responsivity and FWHM are attributed to the observed roughness in the tuning layer. This roughness is visible in the optical microscope image of Fig. 2b and has a standard deviation of ~25 nm for the thickest tuning layers. Besides, in the fabrication process of the presented detector arrays a quarter of a 2″ InP wafer was used, resulting in a non-uniform height profile across the sample, likely producing a difference between the measured thicknesses and the ones of the tested device. A strong improvement of the uniformity (e.g., for the resist spinning) can be obtained using full 2″ InP wafers bonded on a Si substrate, resulting in a reproducible fabrication process and thereby a better match with simulations in terms of resonant wavelengths.

### Characterization of the responsivities in the sensing module

The filter arrays used in the sensing experiments are electrically accessible via wire-bonded contacts, so no electrical probes are required for readout. The spectral responsivity *R*_*i*_(*λ*) of each pixel can be obtained in a similar fashion as for the single devices (see Methods Single-pixel response function characterization), however, the readout is now performed using the developed ADC amplifier board. Supplementary Fig. 5 shows the dark-corrected ADC value as a function of the input power incident on the measured pixel. A laser with a fixed wavelength close to the resonance wavelength (at 1315 nm) was used for illumination, in combination with an attenuator to cover a broad range of input powers. For each power twenty measurements, each with an integration time of 512 ms, were taken, from which the average value and the standard deviation were calculated. The ADC value as a function of input power is close to linear over five orders of magnitude, as follows from the fitted exponent of 1.0978 (Supplementary Fig. 5). For small powers, the standard deviation σ in the ADC signal is ~20 ADC, which is limited by the electronic board. In order to determine the minimum measurable optical power, we fitted the responsivity (ADC value per Watt) for low input powers (see inset Supplementary Fig. 5). The ratio of standard deviation to responsivity provides a value ~1.1 pW for the incident power which gives an ADC signal equal to the noise. The reverse bias currents of the detector structures are below 100 nA for an applied voltage between 0 and −1 V.

A collimator (NA = 0.53, and anti-reflection coating 1050–1620 nm) was used in such a way that the illumination spot covers the full array, allowing for simultaneous measurements on all pixels. The position of the collimator was fixed, ensuring a stable illumination condition in all experiments even for a non-homogenous spot, which is of high importance during the sensing experiments. Note that the characterization of the array is not required for the actual sensing experiment but is merely to check the sensor after wire-bonding; the sensing is directly performed on the measured photocurrent values without the need for spectral reconstruction. The responsivities for the wire-bonded array used in the presented sensing experiments are given in Fig. 4b. Note that this is a different array with different tuning layer heights as shown in Fig. 2c. In addition, this array is characterized using the fiber-coupled collimator with a different NA as employed in the characterization setup for individual pixels. The difference in the optical response between 850 and 1050 nm can be explained by the anti-reflection coating present on the collimator.

### Measurement procedure raw milk samples

Commercially available long-term raw milk standards were used, containing calibrated fat concentrations varying between 2.02 and 6.02 g/100 g (QSE GmbH, Germany). The frozen samples were heated in a water bath at 43 °C for 50 min to defrost and completely dissolve all the fat, resulting in homogenous emulsions. After cooling down, four original samples were pair-wise mixed to increase the number of samples to 16, following the scheme proposed in ref. ^42^ (see Supplementary Fig. 6). The four unmixed standards (F1, F2, F7, and F8) were divided over two containers each and another fully calibrated standard (F3) was added to the dataset and divided over three containers, resulting in a total of 23 milk samples with 17 different fat concentrations. The milk samples were kept in a water bath set to 24 °C (equal to the ambient temperature) to minimize variations in the transflection spectra due to temperature fluctuations. The transflection spectra were measured using a fiber-coupled dip probe (TP300-VIS-NIR, Ocean Insights). The path length of 2 mm, resulting in transmittance of around 1%, is close to the optimal in the NIR range due to strong light scattering on the fat globules in combination with water absorption in this range^43^. To ensure that sufficient light reaches our detector array, a 20 W halogen light source was used as an illumination source (HL-2000-HP, Ocean Insights, 8.8 mW average output power). The output channel of the probe was directly connected to the collimator using multimode fiber (400 µm core) to focus the light on the full sensor array—this is the same system used in the calibration of the arrays (see Methods Characterization of the responsivities in the sensing module).

For each measurement, the average of ten acquisition cycles performed by the sensor was taken, resulting in 16 ADC photocurrent values. One of the 16 channels was disconnected to correct for the drift in the amplifier board. Three dark current measurements were performed with the transflection probe in the air and the illumination light source turned off. Each milk sample was measured three times. Additionally, three reference measurements in the standard illumination condition were taken with an empty probe. Air is taken as a reference since in the current configuration there is a mirror positioned behind the sample. The photocurrent values measured from samples with varying fat concentrations were corrected for the dark current and drift. In this procedure, first, the dark current ADC values were subtracted from the samples’ measured ADC signal for each channel respectively. Then the residual value of the disconnected channel was used for drift correction of the remaining 15 channels. As the drift is the same for every channel, the residual value of the disconnected channel indicates the present drift in the amplifier board. The corrected values were then transformed into scaled absorbance: log_10_(*I*_*r*_/*I*_s_), where *I*_*r*_ and *I*_*s*_ represent the measured photocurrents of the reference and milk samples, respectively. Note that in this procedure the *I*_*r*_ of the reference signal is not corrected for the dark current and therefore this scaled absorbance does not provide an absolute absorbance value.

### PLS analysis on measured photocurrent values

Eight fat samples were assigned to the test set (see filled circles Supplementary Fig. 6) and the remaining nine were used for training the prediction algorithm, following the procedure of^42^. The 15 scaled absorbance values from each measurement of the calibration sample set were mean-centered and then directly used in building prediction models to predict the concentration of fat. Outlier analysis was performed using *Q* residuals and Hotelling’s *T*^2^, which indicates the variation remaining in each sample after projection through the model and the distance between each sample and the multivariate mean within the model, respectively^44^. Measurements with abnormally high variance from the expected means and not belonging to samples containing the highest or lowest fat concentrations were identified as outliers. Two out of 70 measurements of the entire sample set were identified as outliers and excluded from subsequent analysis.

To model fat concentration using scaled absorbance values from our sensor measurements, partial least squares regression (PLS)^44^ was used to reduce the multidimensional pixel data to latent variables (LVs), from which a regression model was built for the prediction of fat content. Eight samples were assigned to the test set, while the remaining nine fat concentrations were the calibration set used to train the prediction algorithm (see Fig. 4d). The PLS algorithm was implemented in Python using packages from NumPy^45^, Matplotlib^46^, and Scikit-learn^47^. The regression models were evaluated based on leaving one group out cross-validation, where replicate measurements from each sample were kept together in one group. To optimize the PLS regression models, the variations of RMSE of calibration (RMSEC) and RMSECV were examined for increasing numbers of LVs (Supplementary Fig. 7a). The subset of LVs whose contributions appreciably decreased the RMSECV without making the RMSECV diverge strongly from the RMSEC were identified and used in the subsequent evaluation of the test sample set.

The statistical measures used to assess the prediction performance of the models included RMSE from both the calibration (RMSECV and RMSEC) and test set (RMSEP), the coefficient of determination (*R*^2^), and the ratio of performance to standard deviation (RPD) (see Supplementary Table 1). Interestingly, only five LVs are required to give *R*^2^ = 0.92 and an RPD value of 3.57 (see Fig. 4d and Supplementary Fig. 7c), indicating an accurate prediction model.

### PLS analysis on full NIR spectra

Equivalent modelling and validation procedures as described in the Methods for the pixel array were also applied to the normalized full absorbance spectra of the milk samples. The commercially available mini spectrometer (Avantes, AVASPEC-MINI-NIR256-1.7) measures in the range from 975 to 1670 nm in 1 nm steps resulting in 696 data points per measured fat sample. PLS was applied to reduce the multidimensional spectral data to LVs from which a regression model was built for the prediction of fat content. The loading plot in Supplementary Fig. 8 illustrates the contribution of each variable (i.e., wavelength) to the three most significant LVs and highlights the spectral regions relevant for the measurement of fat. We observed that the three illustrated LVs were sufficient to explain 97.2% of the variance in the fat concentration, as calculated from the *R*^2^ values. The LVs spectral functions are broad, indicating that a reduced number of filters with limited resolution can be adequate for the sensing problem at hand. Supplementary Fig. 7b shows the RMSEC and RMSECV as a function of the number of latent variables. The predicted fat content as a function of the measured fat is shown in Supplementary Fig. 7d, resulting in *R*^2^ = 0.94 for three LVs.

## Supplementary information

Supplementary Information

## Data Availability

The data that support the findings of this study are available from the corresponding author upon reasonable request. Source data are provided with this paper.

## Source data

Source Data
