# Supplementary material for: Integrated near-infrared spectral sensing
Source: Nat Commun. 2022 Jan 10;13:103. doi: 10.1038/s41467-021-27662-1 (PMC8748443; doi:10.1038/s41467-021-27662-1)
Supplement: Supplementary file 1 — Supplementary Information [file 41467_2021_27662_MOESM1_ESM.pdf]

SUPPLEMENTARY INFORMATION for

Integrated Near-Infrared Spectral Sensing

Kaylee D. Hakkel[1†*], Maurangelo Petruzzella[1†], Fang Ou[1], Anne van Klinken[1], Francesco Pagliano[1], Tianran Liu[1], Rene P.J. van Veldhoven[1], Andrea Fiore[1].

[1] *Department of Applied Physics and Institute for Photonic Integration, Eindhoven University of Technology, PO Box 513, NL 5600 MB Eindhoven, The Netherlands.*

[†] These authors contributed equally to this work

[*] Correspondence should be addressed to k.d.hakkel@tue.nl

Supplementary Discussion 1. Classification of different plastic types

The plastic samples used in this study were retrieved by members of the research group from their typical household waste. 48 samples were used in total, with 13 samples assigned to the test set while the remaining 35 were used to train the classification algorithm. The samples included different thicknesses, colours and texture, belonging to types: 1 (PET), 4 (LDPE), 5 (PP) and 6 (PS) (see Supplementary Fig. 9a).

The plastic samples, placed on top of standard white printing paper, were illuminated with a 20 W halogen light source (HL-2000-HP, Ocean Insights, 8.8 mW average output power) via a fibre coupled reflection probe (QR400-7-VIS-NIR, Ocean Insights), which is used to measure the specular reflectance and backscatter from the samples. The output channel of the probe was directly connected to the collimator using a multimode fibre (400 µm core) to focus the light on the full sensor array - this is the same system used in the calibration of the arrays (see Methods). The samples were measured three times at approximately the same position. Each measurement yields 15 photocurrent values, which is averaged from approximately five acquisition cycles performed by the sensor. The photocurrents from each measurement were dark-corrected and then drift-corrected by subtracting the measurements from a selected pixel. Thus, the resulting sensor measurement consists of corrected photocurrent values for 14 pixels. Note that the same RCE array is used as for the milk experiments (see Fig. 4b), although we made use of only 15 pixels. Reflectance signals acquired directly from the white printing paper were used as reference. The corrected photocurrent values from the sensor were converted to normalized photocurrent values: $\log_{10}(I_r/I_s)$, where I_r and I_s represent the measured intensity of the reference and plastic samples, respectively. We note that this measurement and normalization method produces a set of photocurrents which depend on both the transmittance and reflectance values of the plastic.

The 14 normalized photocurrent values from each measurement (see Supplementary Fig. 9c) were pre-processed by dividing the individual scaled absorbance values by the sum of the 14 channels. The strong variation in intensity for different plastic samples within a single type is attributed to the differences in colour and thickness, which both strongly influence the reflectance characteristics. The pre-processed normalized photocurrents were mean centred and directly used in PLS combined with linear discriminant analysis (PLS-LDA) to build a classification model for predicting the plastic type. The PLS-LDA procedure consists of a PLS dimension reduction and subsequent LDA applied on the PLS LVs[1]. The number of LVs was optimized based on the model which resulted in the highest prediction accuracy in groupwise cross-validation. The optimized PLS-LDA model was used for subsequent evaluation of the test dataset. Equivalent modelling and validation procedures were also applied to the normalized signals measured by the mini

spectrometer (Avantes, AVASPEC-MINI-NIR256-1.7) (see Supplementary Fig. 9b). The modelling and optimization algorithms were implemented in Python using packages from NumPy[2], Matplotlib[3] and Scikit-learn[4].

The optimized prediction model of the sensor data used 13 LVs in the LDA classifier, which achieved 89.6% accuracy in cross-validation (see Supplementary Fig. 9d) and 94.9% in prediction of the test samples (see Supplementary Fig. 9e). Those values are comparable to prediction accuracies obtained for commercially available miniaturized spectrometer solutions, where the full measured spectra in the 1400 to 2500 nm range are used to build a prediction model for the plastic type[5]. In comparison, the optimized prediction model of the commercial spectrometer data used 4 LVs in the LDA classifier, which achieved 100% accuracy in both cross-validation and prediction of the test samples. The slight improvement in accuracy obtained using the commercial spectrometer may be attributed to its ability to clearly resolve the characteristic spectral features of each plastic type. As shown in see Supplementary Fig. 9b, the characteristic spectral features that distinguish between the plastics are quite narrow compared to the RCE filter response. However, we anticipate that the prediction performance of the sensor data will further improve with optimization of the RCE filter design using similar methods as shown for the milk sensing (see Supplementary Discussion 2).

Supplementary Discussion 2. Optimization of the RCE structures for sensing

Higher prediction accuracies can be obtained via improvement of both the fabrication process and optimizing the design of the multi-pixel array. This can be confirmed from the simulations performed on the experimental transmittance data of 300 raw milk samples with fat percentages from 2.7% to 7.9%, as reported in[6]. These milk samples were measured in transmission using a silica cuvette and the spectra were acquired using an ASD LabSpec Pro spectrophotometer containing a Si diode array and two Peltier cooled InGaAs detectors, resulting in lower noise levels as compared to the spectra measured with the mini spectrometer used in this article (see Fig. 3b). The expected photocurrent values were calculated by taking the spectral integral of the detector response curves $R_i(\lambda)$ multiplied by the transmission spectrum $S(\lambda)$ of the milk samples, as expressed by Eq. 1.

Using this method, the expected prediction accuracy of the fabricated array can be calculated for a larger dataset. To simulate this sensing experiment, the measured response curves of Fig. 4b were used, where pixel 6 (e.g. 170 nm tuning layer) is discarded as employed for the drift correction in the sensing experiment. The 15 photocurrent values for each milk sample were used to build a PLS regression model, using the same method as explained in the Methods. No outlier selection on the transmission spectra was performed in this case. 12 LVs were required to give a coefficient of determination R^2 of 0.94 and RPD value of 4.2, showing an improvement of the prediction accuracy compared to our sensing experiment where only 16 different milk samples were measured. Note that there was no division between test and validation set in this analysis; instead Monte-Carlo cross-validation was performed for 100 iterations, with 20% of the entire dataset used for validation.

Further improvement of the prediction accuracy can be achieved by optimizing the tuning layer thicknesses of all 15 pixels for the specific application. To improve the computational speed, the RCE detectors were described using a simple 1D model[7], based on the transfer matrix method, instead of using the time-consuming FDTD simulations. To avoid the complications of PLS analysis, a simpler multiple linear regression (MLR) model was used. Since the photocurrent values are directly implemented in the model, the use of MLR allows for faster optimization of the

prediction accuracy while sweeping the thicknesses of all 15 tuning layers. Using a 10 nm Au top mirror for all pixels fixes the linewidth of the optimized resonances. By optimizing the tuning layer thicknesses, a significantly higher prediction accuracy of the fat content in milk was achieved, resulting in R^2=0.98 and an RPD of 7.63, matching the prediction accuracies for fat obtained in Ref. [6] in the same wavelength range.

These optimization results demonstrate that our sensing approach can provide a prediction performance comparable to the best results obtained with state-of-the-art conventional spectrometers. We note that implementing an arbitrary set of thicknesses in the array is straightforward using grey-scale lithography, and that different sets, optimized for different application cases, can be included in a single fabrication process.

Supplementary Discussion 3. Sugar sensing in tomatoes

To show the ability of measuring more complex spectral signatures using the fabricated spectral sensor, we performed the analysis on sugar in tomatoes. An experimental dataset with the reflectance spectra of 100 tomatoes, was provided by the Wageningen University & Research, following the same measurement approach as explained in [8,9]. The expected photocurrents corresponding to tomatoes with different sugar concentrations are calculated using the measured response curves of Fig. 4b and Eq. 1, following the procedure as explained in section ST2. Pixel 6 (e.g. 170 nm tuning layer) is discarded as employed for the drift correction in the sensing experiment. The 15 photocurrent values for each tomato were used to build a PLS regression model, using the same method as explained in Materials and Methods section PLS analysis on measured photocurrent values. No outlier selection on the reflectance spectra is performed in this case. 14 LVs were required to give a coefficient of determination R^2 of 0.92 and RPD value of 3.83. Note that there was no division between test and validation set in this analysis; instead Monte-Carlo cross-validation was performed for 100 iterations, with 20% of the entire dataset used for validation.

The prediction accuracy can be further improved by optimizing the tuning layer thicknesses of the 15 pixels, in a similar way as it was shown for the milk sensing experiments in the main paper and ST2. A 10 nm thick Au mirror is used for all pixels and thereby fixes the linewidth of the filter responses. Using the 1D model of resonant cavity enhanced detectors[10] to calculate the filter response, in combination with a MLR algorithm, we design an optimized array which gives an R^2 of 0.95 and RPD value of 5.09. This RPD value above 3 is generally accepted to indicate a properly working prediction model[11], showing the capability of our spectral sensing array to measure more complex spectral signatures like sugar in tomatoes. These results are comparable to prediction performances obtained with conventional spectrometers. We note that implementing an arbitrary set of thicknesses in the array is straightforward using grey-scale lithography, and that different sets, optimized for different application cases, can be included in a single fabrication process.

References

1. Boulesteix, A.-L. PLS Dimension Reduction for Classification with Microarray Data. *Statistical Applications in Genetics and Molecular Biology* **3**, 1–30 (2004).
2. Oliphant, T. E. *Guide to Numpy*. (CreateSpace Independent Publishing Platform, 2015).
3. Hunter, J. D. Matplotlib: A 2D Graphics Environment. *Computing in Science & Engineering* **9**, 90–95 (2007).
4. Pedregosa, F. *et al.* Scikit-learn. *GetMobile: Mobile Computing and Communications* **19**, 29–33 (2015).
5. Schmidt, F., Christiansen, N. & Lovrincic, R. The Laboratory at Hand: Plastic Sorting Made Easy. *PhotonicsViews* **17**, 56–59 (2020).

6. Aernouts, B., Polshin, E., Lammertyn, J. & Saeys, W. Visible and near-infrared spectroscopic analysis of raw milk for cow health monitoring: Reflectance or transmittance? *Journal of Dairy Science* **94**, 5315–5329 (2011).
7. Ünlü, M. S. & Strite, S. Resonant cavity enhanced photonic devices. *Journal of Applied Physics* **78**, 607–639 (1995).
8. Polder, G. & Heijden, G. van der. Measuring Ripening of Tomatoes Using Imaging Spectrometry. in *Hyperspectral Imaging for Food Quality Analysis and Control* 369–402 (Elsevier, 2010). doi:10.1016/B978-0-12-374753-2.10012-7.
9. G. Polder, G. W. A. M. van der Heijden & I. T. Young. Spectral Image Analysis for Measuring Ripeness of Tomatoes. *Transactions of the ASAE* **45**, 1155–1161 (2002).
10. Kishino, K. *et al.* Resonant cavity-enhanced (RCE) photodetectors. *IEEE Journal of Quantum Electronics* **27**, 2025–2034 (1991).
11. Li, X., Xu, Z., Cai, W. & Shao, X. Filter design for molecular factor computing using wavelet functions. *Analytica Chimica Acta* **880**, 26–31 (2015).

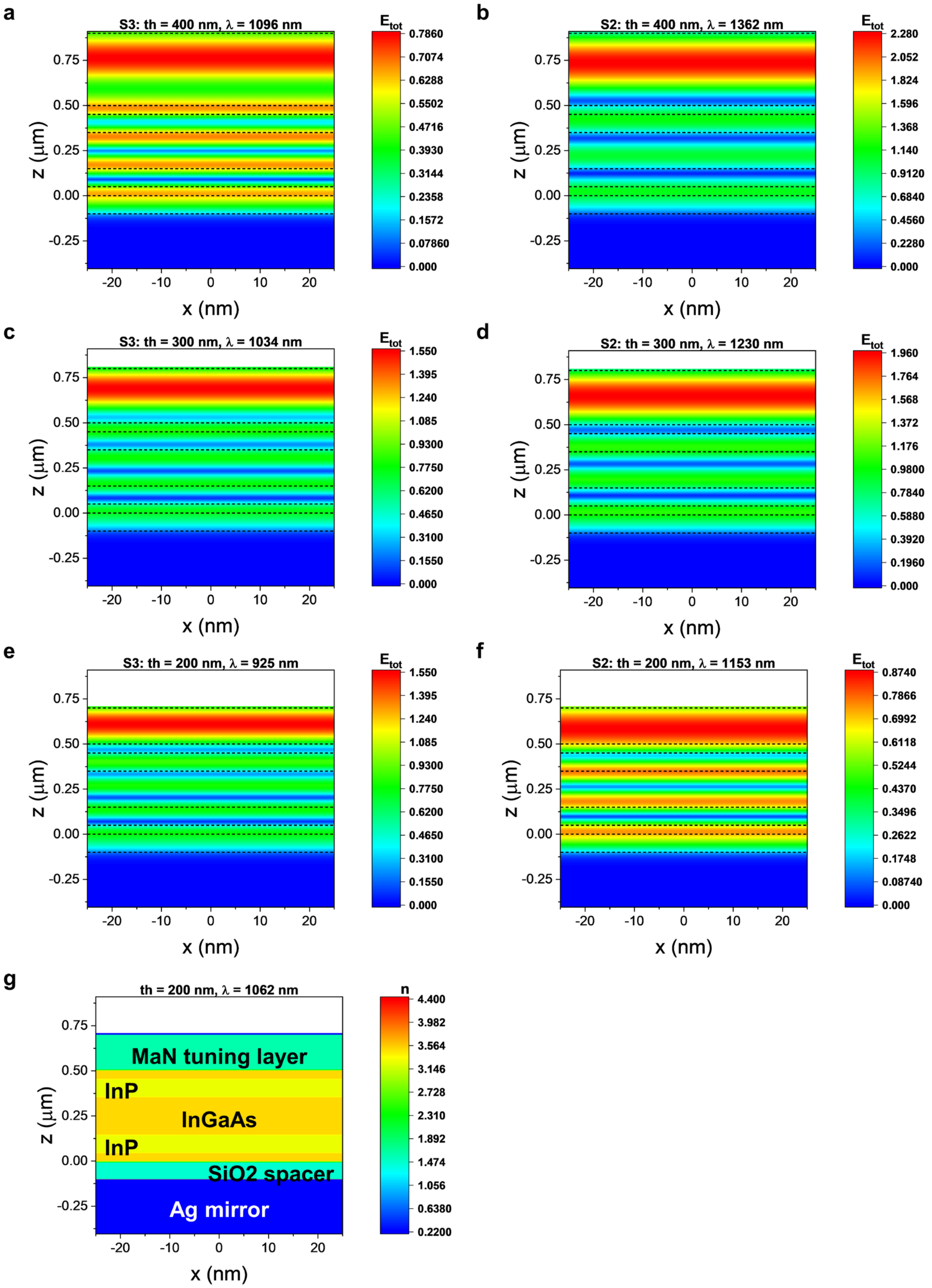

Supplementary Fig. 1 Simulated electric field distribution. The simulated modulus of the electric field (*E*) for mode S3 **(a**,**c**,**e)** and S2 **(b**,**d**,**f)** for different thicknesses of the tuning layer. The top Au mirror is 10 nm thick. **g**, Refractive index distribution across the structure at λ=1062 nm for a 200 nm tuning layer. Dashed lines in **a-f** indicate the different materials as indicated in **g**.

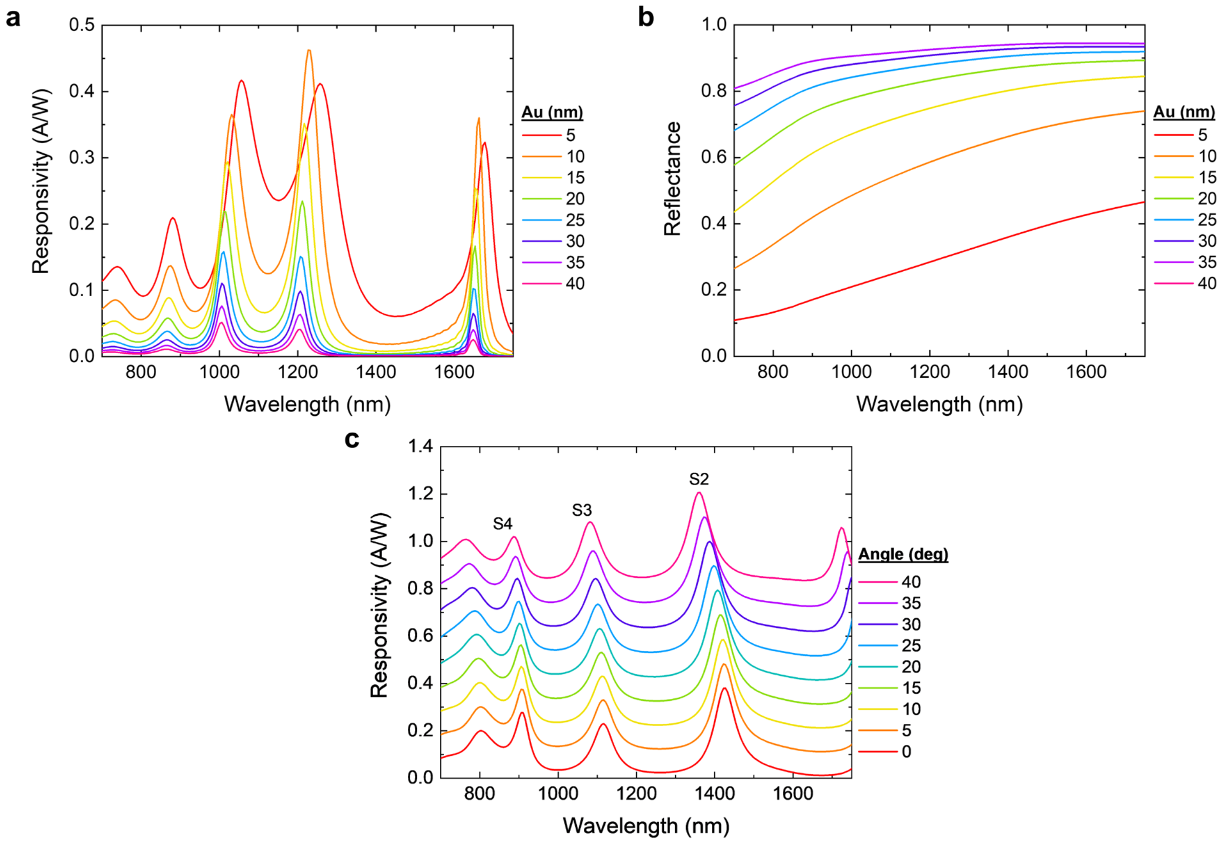

Supplementary Fig. 2 Simulated optical properties of resonant-cavity-enhanced detectors. a, Simulated optical response for different thickness of the top Au mirror for a 300 nm tuning layer. **b**, Reflectance of the Au mirror for a wave incident from the ma-N layer for different thicknesses of the Au layer. **c**, Simulated angular dependence for a structure with a 450 nm tuning layer and 10 nm top Au mirror. An offset is added for clarification.

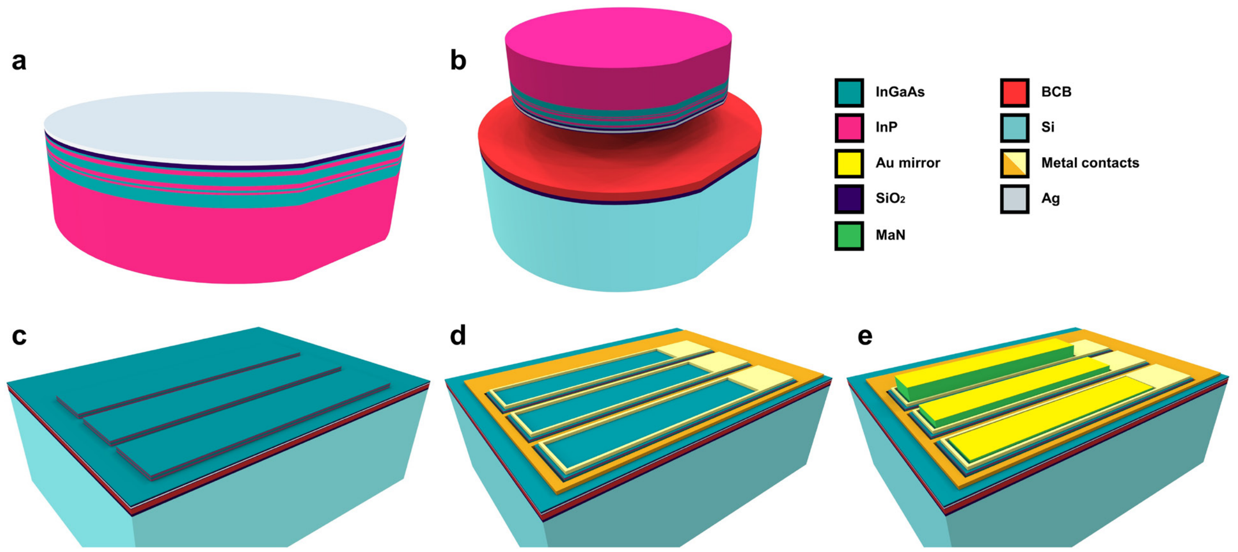

Supplementary Fig. 3 Schematic representation of the fabrication process. a, InP wafer with epitaxially grown InP and InGaAs layers, the deposited SiO_2 space layer and the Ag mirror. **b**, InP wafer positioned above the Si wafer for adhesive wafer bonding. **c**, Definition of the mesa structures with common p-contact layer. **d**, Fabrication of the p- and n- metal contact pads. **e**, Three-dimensional height profile generated by grey scale lithography followed by the deposition of the top Au mirror.

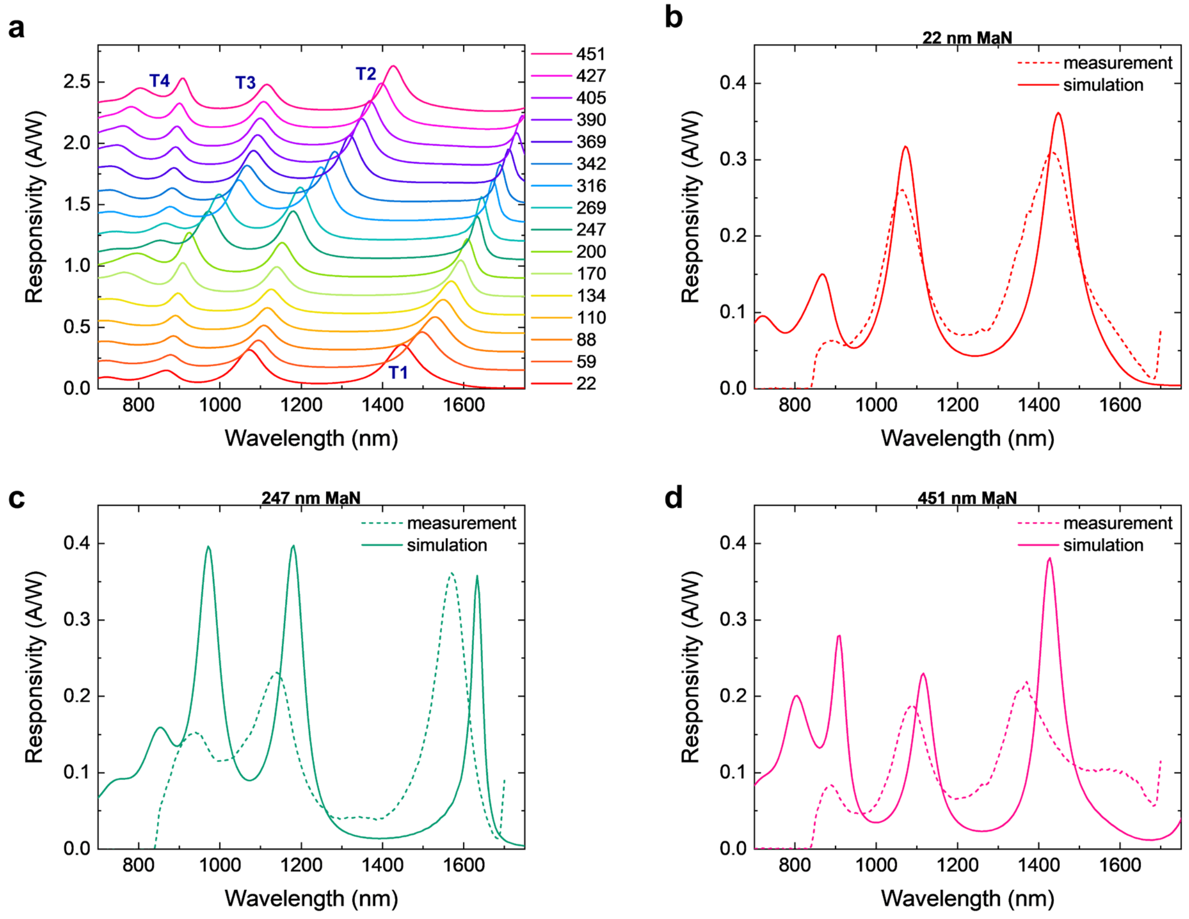

Supplementary Fig. 4 Simulated response curves for fabricated tuning layer heights. a, Simulated responsivity of the RCE detectors with tuning layer heights the same as in the measured device. **b-d**, Comparison between measured (dashed) and simulated (full) response curves for three different tuning layer thicknesses.

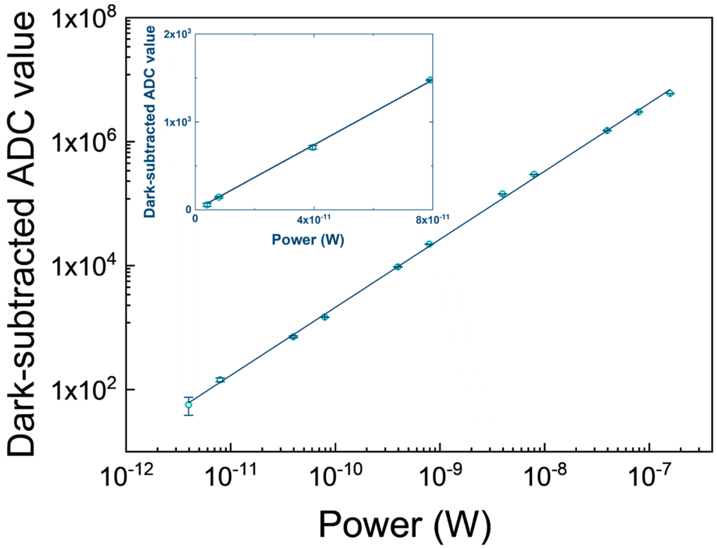

Supplementary Fig. 5 Characterization of the electronic read-out board. The dark corrected ADC values are measured for different input laser powers. The standard deviation σ in the ADC value is indicated by the error bars. For low laser powers the noise level is limited by the electronic board. Solid line indicates the fitting curve $\mathbf{ADC = 2 \cdot 10^{14} \cdot \boldsymbol{P}^{1.098}}$. **Inset:** ADC values for low input powers with fitted slope $\mathbf{1.85 \cdot 10^{13}}$ ADC/W.

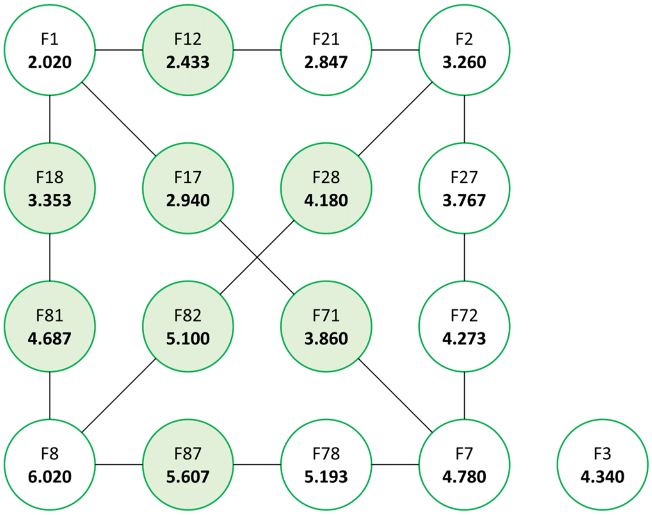

Supplementary Fig. 6 Mixing scheme of milk samples. 4 samples (F1, F2, F7, F8) with calibrated fat content (g/100 ml) were pair-wised mixed to obtain 16 different fat concentrations. A fully-calibrated sample F3 was also added to the calibration set. Filled circles indicate the test sample set.

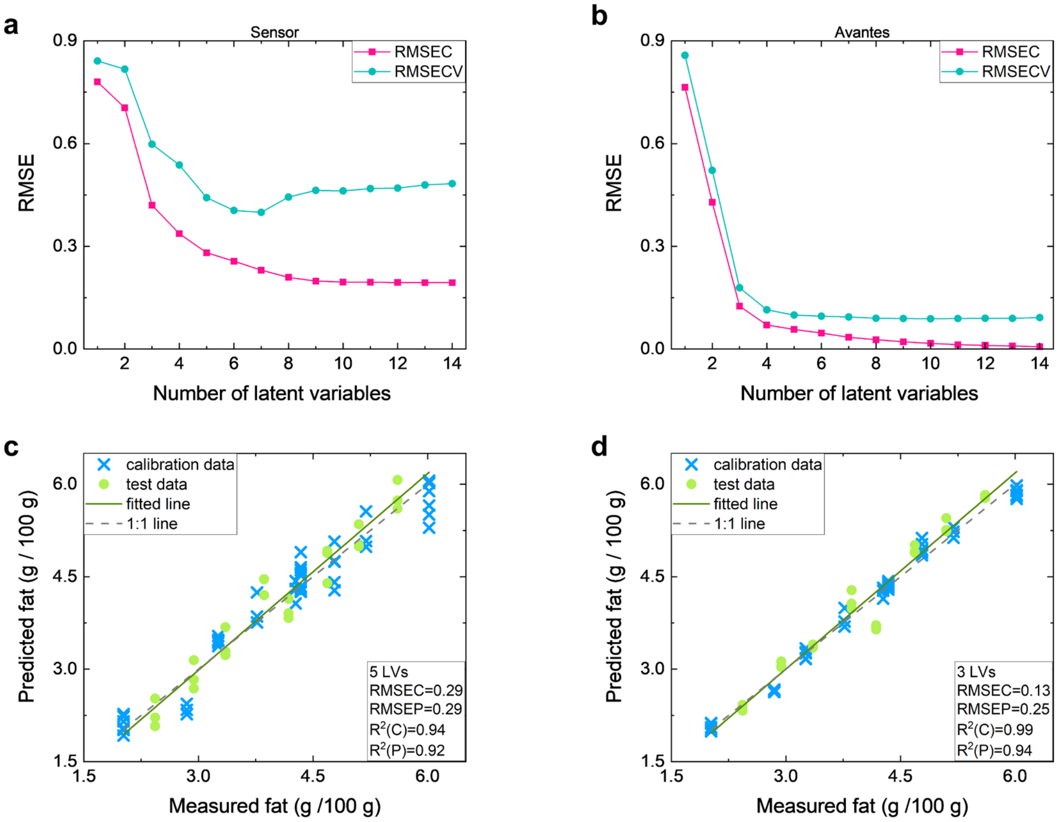

Supplementary Fig. 7 PLS regression models of the sensor and spectrometer data. The RMSECV and RMSEC vs number of PLS components for the sensor data **(a),** and spectrometer data **(b)**. The optimised number of components for the PLS regression model was 5 and 3 for the sensor and spectrometer data, respectively. The fat content was predicted via PLS models built using sensor **(c)** and spectrometer **(d)** data and is compared to the expected fat content. Note that **(c)** is the same graph as shown in Fig. 4d, however is added for comparison.

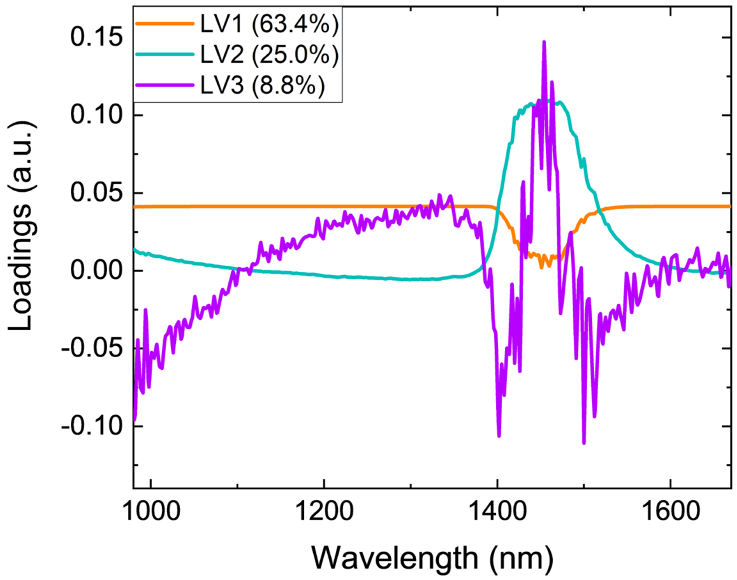

Supplementary Fig. 8 PLS loadings plot of the full spectral data. PLS loadings of the first three latent variables (LVs) plotted as function of wavelength. The values in brackets indicate the proportion of variance explained in fat concentrations by each latent variable.

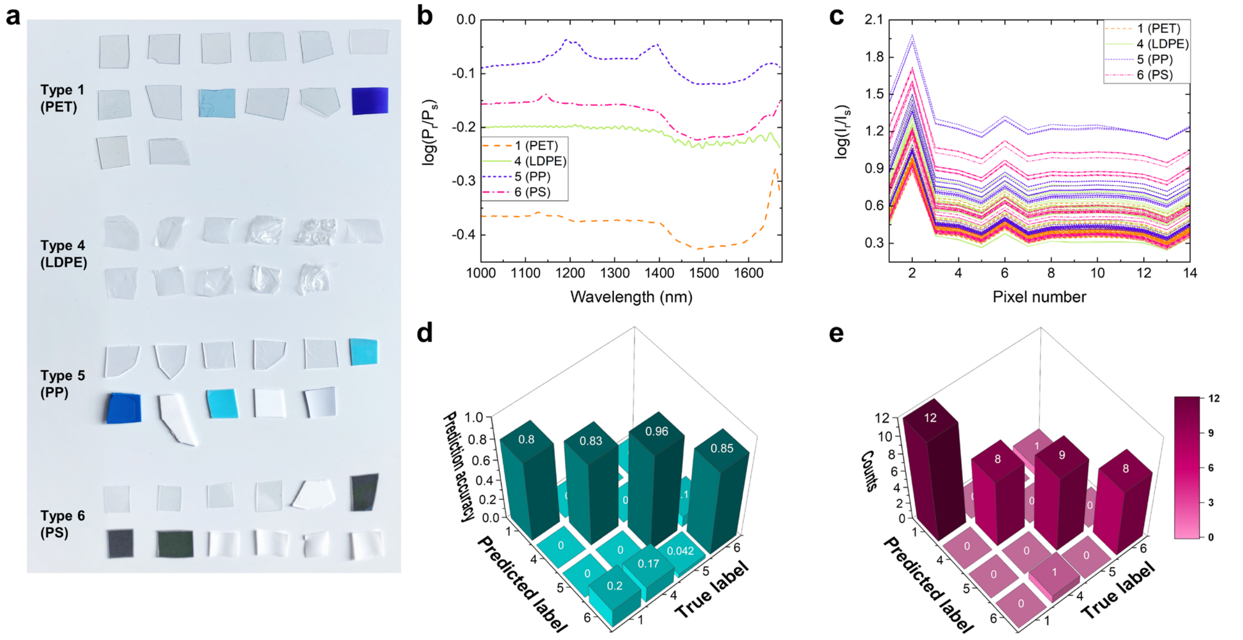

Supplementary Fig. 9 Spectral sensing of plastic samples. a, Measured plastic samples of four different types. **b**, Averaged normalized signals $\mathbf{log_{10}}(\boldsymbol{P_r/P_s})$ of the four different plastic types, measured by a commercial spectrometer. **c**, Normalized photocurrents $\mathbf{log_{10}}(\boldsymbol{I_r/I_s})$ measured with an RCE detector array. **d**, Relative confusion matrix from internal cross-validation using the calibration dataset, with an accuracy of 89.6%. **e**, Absolute confusion matrix from the prediction of the test dataset, with an accuracy of 94.9%.

Supplementary Table 1. Model statistics for the prediction of fat content.

			Cross-validation	Validation on test data						
**Device**	**no. of variables**	**no. of LVs[1]**	**RMSECV[2]**	**RMSEP[2]**	**SEP[3]**	**R^2**	**RPD[4]**	**Bias[5]**	**Slope**	**Offset**
**RCE detector array**	15	5	0.44	0.29	0.29	0.92	3.57	7.77×10^{-16}	1.06	-0.21
**Avantes mini-spectrometer**	696	3	0.18	0.25	0.25	0.94	4.05	-1.09×10^{-16}	1.06	-0.18

For both measurement methods, analysis was performed using PLS regression.
[1] LVs = latent variables.
[2] RMSECV and RMSEP = root mean square error of cross-validation (CV) and prediction (P), expressed in fat percentages (g/100 g).
[3] SEP = standard error of prediction, expressed in fat percentages (g/100 g).
[4] RPD = ratio of performance to deviation.
[5] The bias is calculated for the validation based on the calibration dataset.
